# Stroma-derived but not tumor ADAMTS1 is a main driver of tumor growth and metastasis

**DOI:** 10.18632/oncotarget.8922

**Published:** 2016-04-22

**Authors:** Rubén Fernández-Rodríguez, Francisco Javier Rodríguez-Baena, Estefanía Martino-Echarri, Carlos Peris-Torres, María del Carmen Plaza-Calonge, Juan Carlos Rodríguez-Manzaneque

**Affiliations:** ^1^ GENYO, Centre for Genomics and Oncological Research, Pfizer/Universidad de Granada/Junta de Andalucía, Granada 18016, Spain

**Keywords:** extracellular protease, extracellular matrix, hypoxia, tumor stroma, vasculature

## Abstract

The matrix metalloprotease ADAMTS1 (*A Disintegrin And Metalloprotease with ThromboSpondin repeats 1*) has been involved in tumorigenesis although its contributions appeared ambiguous. To understand the multifaceted actions of this protease, it is still required a deeper knowledge of its implication in heterogeneous tumor-stroma interactions. Using a syngeneic B16F1 melanoma model in wild type and ADAMTS1 knockout mice we found distinct stroma versus tumor functions for this protease. Genetic deletion of ADAMTS1 in the host microenvironment resulted in a drastic decrease of tumor growth and metastasis. However, the downregulation of tumor ADAMTS1 did not uncover relevant effects. Reduced tumors in ADAMTS1 KO mice displayed a paradoxical increase in vascular density and vascular-related genes; a detailed characterization revealed an impaired vasculature, along with a minor infiltration of macrophages. In addition, ex-vivo assays supported a chief role for ADAMTS1 in vascular sprouting, and melanoma xenografts showed a relevant induction of its expression in stroma compartments. These findings provide the first genetic evidence that supports the pro-tumorigenic role of stromal ADAMTS1.

## INTRODUCTION

The impact of the communication between cancer cells and stroma constituents during distinct stages of tumor progression has been an inspiring field of investigation during the last decades. Multiple efforts have highlighted the contribution of stroma cells such as cancer-associated fibroblasts, endothelium, macrophages and other immune-related populations, with major consequences to the design of therapeutic tools. Still, the multitude of factors contributing to this complex scenario of tumor-stroma interactions demands a deeper understanding. For example, studies of extracellular proteases as modifiers of the tumor microenvironment have revealed their participation as oncogenic as well as tumor-protective molecules. In addition to the putative expression of these proteases by both tumor and stromal cells, the presence of specific substrates would further redefine their final effect.

The extracellular protease ADAMTS1 represents a good example of such complexity. Although it was first shown to display anti-angiogenic properties [1], the contribution of ADAMTS1 (and other ADAMTSs) during tumorigenesis is still controversial [2]. Its angiostatic and tumor suppressive properties have been described in distinct models [3–5], but other studies support its relevance in metastasis [6] and tumor growth [7, 8]. As for its catalytic activity it has been observed on various proteoglycans, such as syndecan-4, aggrecan and versican [9–11], and other extracellular components [12–14]. A common finding has been the close connection of ADAMTS1 with neovascularization mechanisms, including its contribution to the acquisition of endothelial-like properties of some tumor cells [5]. Importantly, ADAMTS1 was found to be relevant during endothelial cell sprouting in collagen invasion assays [15], and its expression is induced in endothelial cells under hypoxic conditions and VEGF-treatment [16, 17]. The specific influence of stromal ADAMTS1 has been suggested in some studies of breast cancer [4, 18, 19], and the use of the ADAMTS1 knockout mice, in combination with a spontaneous model of mammary carcinogenesis, revealed its participation in tumor growth and metastasis [7].

Here, to examine the contribution of ADAMTS1 by different cell compartments, we carried out syngeneic tumor experiments in wild type and ADAMTS1 KO mice. Although levels of the protease were still provided by tumor cells, the absence of ADAMTS1 in the host stroma significantly impaired B16F1 tumor growth and metastasis. Our studies also revealed an unexpected effect in the infiltration of macrophage populations. In addition, the downregulation of ADAMTS1 in B16F1 tumor cells was not accompanied by relevant changes in their tumorigenic properties. Finally, a combination of human melanoma xenografts and ex-vivo experiments confirmed the main contribution of the stroma as a supplier of ADAMTS1 and its relevance for vascular functionality.

## RESULTS

### The absence of ADAMTS1 in the host stroma results in tumor growth delay

**Figure 1:**
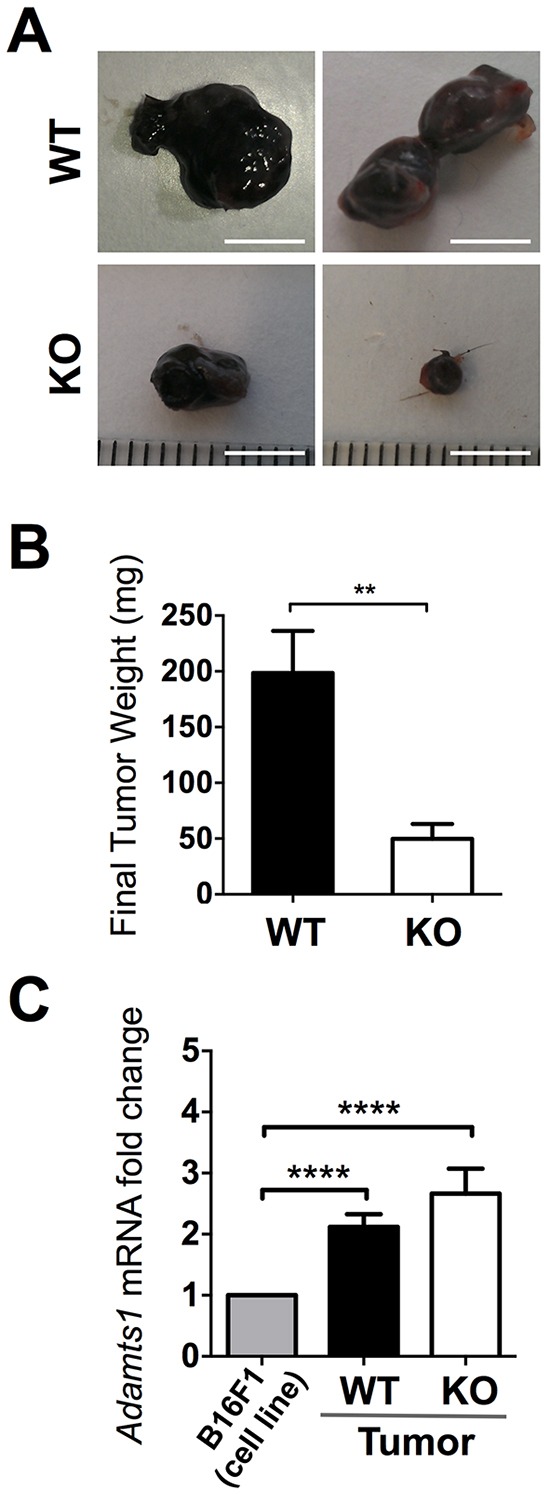
Tumor growth of B16F1 cells in WT and ATS1-KO mice **A.** Images of representative tumors obtained in WT and ATS1-KO mice after 18 days post-injection (Scale Bar= 5 mm). **B.** Graph representing mean tumor weight ± SEM after 18 days post-injection (Number of animals in this experiment: WT, n=7; ATS1-KO, n=6). **C.** Graph representing *Adamts1* gene expression in the generated tumors, in comparison with B16F1 cells in culture. Bars show mean values ± SEM. (**, p < 0.01; ****, p < 0.0001).

To better understand the role of ADAMTS1 during tumor growth and to unveil its matrix-dependent actions, we generated and characterized syngeneic tumors with B16F1 murine melanoma cells in wild type (WT) and *Adamts1* knockout (ATS1-KO) mice. We injected these cells subcutaneously in WT and ATS1-KO mice and followed tumor progression for 18 days. Thereafter, animals were sacrificed and tumors were dissected and thoroughly cleaned of surrounding tissue. The evaluation of tumors revealed a significant reduction of tumor weight in ATS1-KO animals compared to their WT littermates (Figure 1A–1B). According to the endogenous presence of *Adamts1* in B16F1 cells in culture (Supplementary Figure S1), we also evaluated by qPCR its gene expression in the generated tumors. This analysis revealed significant levels of *Adamts1* in the tumor comparing with B16F1 cells in culture; nevertheless no major changes between WT and ATS1-KO mouse tumors were observed (Figure 1C). This result indicates that, although *Adamts1* is relevantly present in the tumor, its absence in the host stroma cells (in the ATS1-KO group) is enough to produce a severe delay in tumor development.

### The absence of ADAMTS1 in the host stroma results in the alteration of the vasculature of tumors

**Figure 2:**
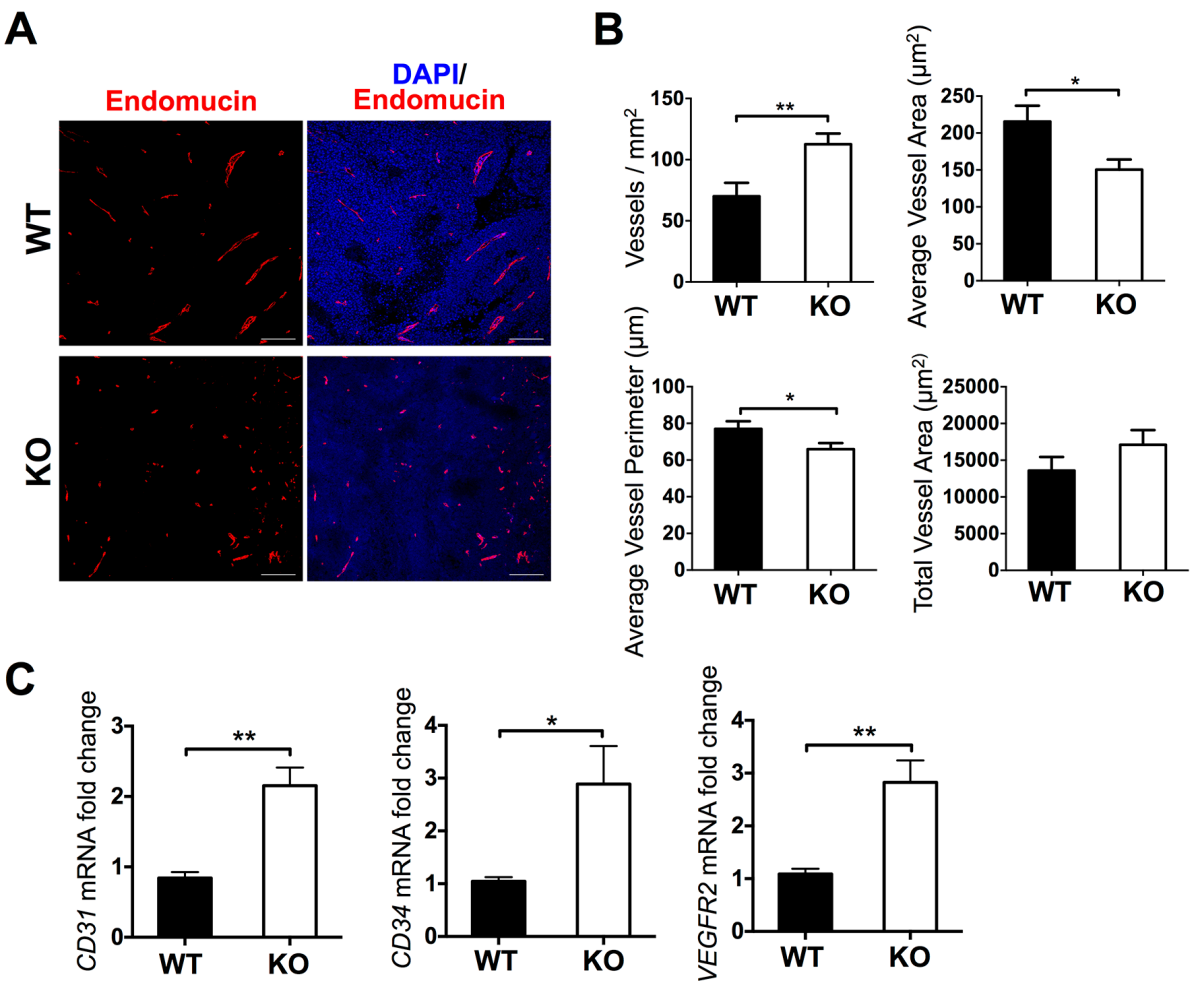
Characterization of vasculature in tumors from WT and ATS1-KO mice **A.** Fluorescence microscopy images of representative sections showing Endomucin and DAPI staining. **B.** Graphs representing results from the morphometric analyses of vasculature (Metamorph 7 software). These analyses include vessel density (vessels/mm^2^), average vessel area (μm^2^), average vessel perimeter (μm), and total vessel area (μm^2^). Bars show mean values ± SEM. Statistical analyses show unpaired t test. **C.** Graphs representing gene expression evaluation of vascular-related genes. Bars show mean values ± SEM. (*, p < 0.05; **, p < 0.01).

As previous tumor studies have shown, the alteration of ADAMTS1 levels is accompanied by changes in the overall tumor structure and consistency, and specifically it has significant effects on the vasculature [4, 5, 20–23]. Therefore we decided to explore the vascular pattern in our model. To achieve such purpose, we performed an extensive series of histopathological and gene expression analyses. First, we approached single staining of paraffin embedded tumor sections with an antibody against the endothelial marker Endomucin [3] (Figure 2A). Metamorph 7 software was used to quantify and characterize tumor vasculature objectively (more details are included in the Materials and Methods section). These analyses revealed clear differences in certain parameters (Figure 2B). A first assessment showed a significant increase in vessel density (vessels/mm^2^) in the ATS1-KO group when both sets of tumors were compared (Figure 2B). This finding correlated negatively with tumor growth rate (Figure 1B). However, additional related parameters, such as average vessel area and average vessel perimeter did correlate positively with tumor size (Figure 2B). Finally, giving the opposite results of vessel density and average vessel area, the measure of the total vessel area displayed no differences (Figure 2B).

According to the changes observed in the vasculature, we completed the study with the expression analysis of endothelial-related genes in tumor lysates, such as CD31 (PECAM1), CD34, and VEGFR2 (KDR) (Figure 2C). Such evaluation indicated that these endothelial genes were also significantly overexpressed in the tumors in ATS1-KO mice, in line with the increased vessel density showed in the previous panel. Yet, this neovasculature does not seem to be properly functional, as tumor size was clearly diminished in ATS1-KO mice (Figure 1B).

### Tumors generated in ADAMTS1 KO mice displayed an increased hypoxic response

**Figure 3:**
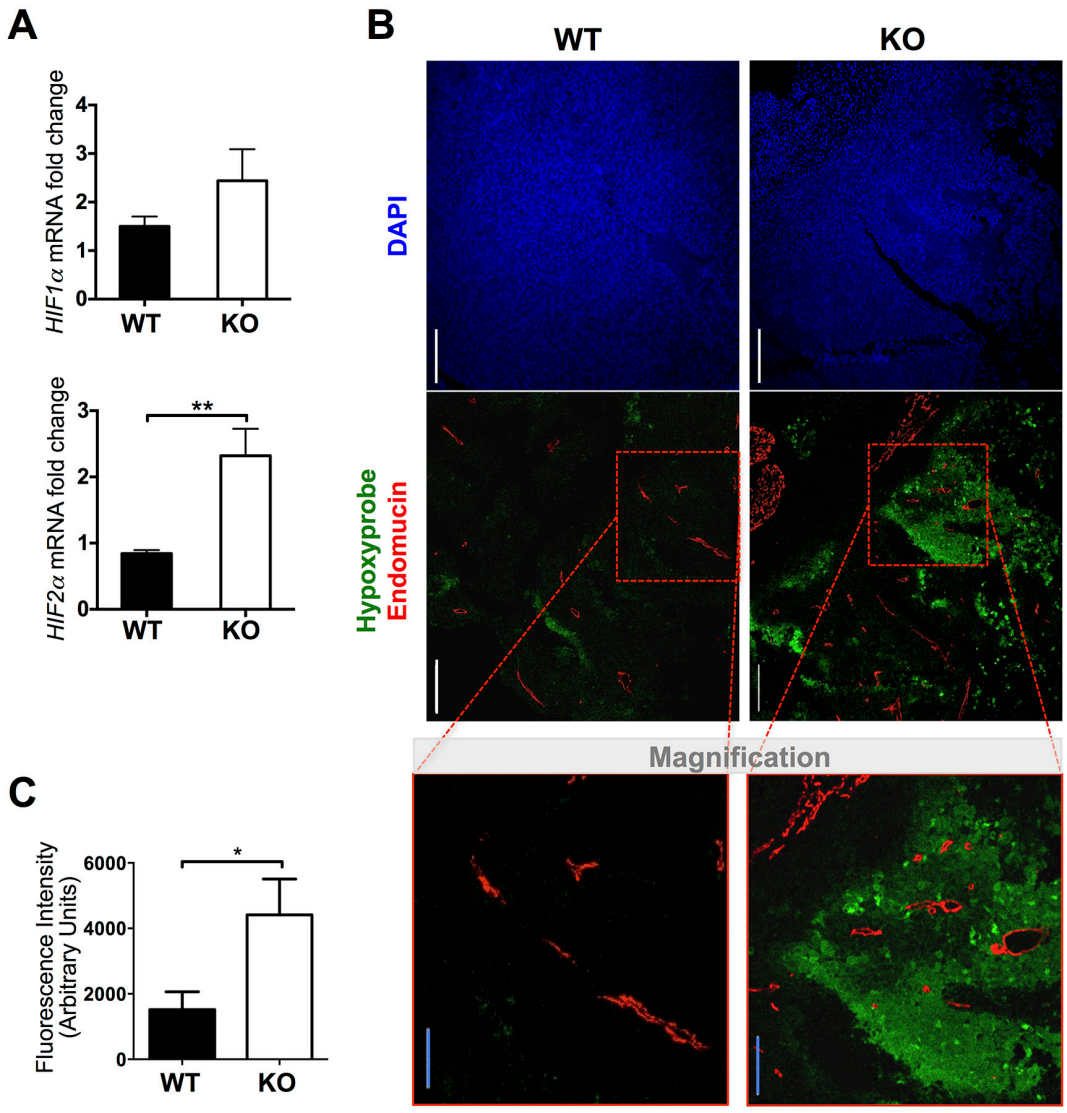
Evaluation of hypoxia in tumors from WT and ATS1-KO mice **A.** Graph representing HIF1α and HIF2α gene expression in the generated tumors. Bars show mean values ± SEM. **B.** Representative images and magnification showing DAPI (blue), Hypoxyprobe (green) and Endomucin (red) immunostaining of tumors sections (scale bars = 200 μm (white bar) and 100 μm (blue bar in magnification)). **C.** Graph representing a quantification of fluorescence intensity in tumor sections from both groups (Number of animals in this experiment: WT, n=6; ATS1-KO, n=6). (*, p < 0.05; **, p < 0.01).

Consistent with the finding of smaller tumors with increased vessel density in ATS1-KO mice, we approached the evaluation of hypoxia as a measure of functionality of the vasculature. First we evaluated the gene expression of hypoxia players HIF1α and HIF2α by qPCR. A significant upregulation was found just for HIF2α in tumors of the ATS1-KO group compared with WT animals (Figure 3A). To verify the existence of hypoxic regions, a group of mice were i.p. injected with a hypoxia probe, Hypoxyprobe, immediately prior to euthanasia [24–26]. Later on, we visualized hypoxic regions in these tumor sections in combination with the immunolocalization of Endomucin-positive vessels (Figure 3B). This assay revealed that little to no hypoxia was found in tumors from WT animals. In contrast, tumors from ATS1-KO mice displayed multiple hypoxic regions. A closer evaluation showed that these zones did not co-localized necessarily with avascular areas or Endomucin-negative regions (Figure 3B). Quantification of hypoxic-related fluorescence intensity (Figure 3C) confirmed that smaller but more abundant vessels in the ATS1-KO tumors are not fully functional, as the oxygen supply seems to be deficient in these tumors.

### Downregulation of ADAMTS1 in tumor cells revealed its minor contribution for tumor progression and angiogenesis

**Figure 4:**
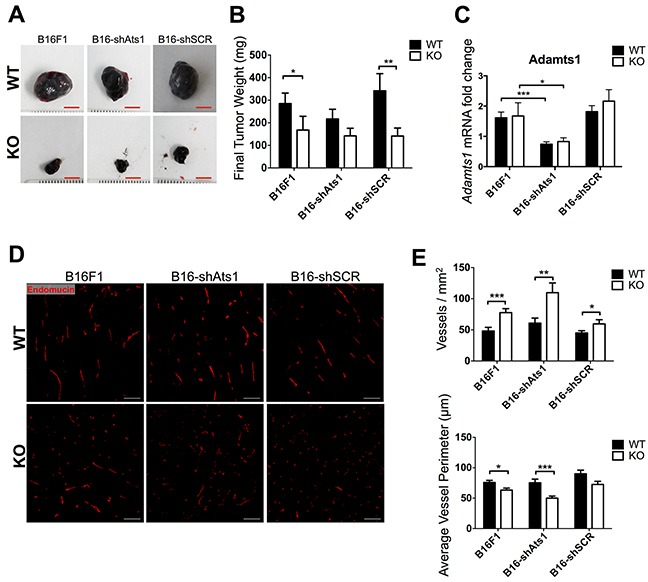
Tumor growth and characterization of vasculature of tumors derived from control and modified B16F1 cells in WT and ATS1-KO mice **A.** Images of representative tumors obtained from different mouse groups after 18 days post-injection (Scale bar= 5 mm). **B.** Graph representing mean tumor weight ± SEM after 18 days post-injection. (Number of animals in this experiment: WT/B16F1, n=17; ATS1-KO/B16F1, n=14; WT/B16-shAts1, n=10; ATS1-KO/B16 shAts1, n=10; WT/B16-shSCR, n=7; ATS1-KO/B16-shSCR, n=7). **C.** Graph representing Adamts1 gene expression in the generated tumors. **D.** Fluorescence microscopy images showing Endomucin staining of tumor sections. Scale bar = 200 μm. **E.** Graphs representing results from the morphometric analyses of vasculature showing vessel density and average vessel perimeter. Bars show mean values ± SEM. Statistical analyses show unpaired Student's t test. (*, p < 0.05; **, p < 0.01; ***, p < 0.001).

At this point, to uncover the contribution of ADAMTS1 provided by tumor cells, we inhibited the expression of endogenous *Adamts1* in B16F1 melanoma cells and we further evaluated their tumorigenic properties in WT and ATS1-KO mice. To obtain a stable downregulation we used shRNA technology that targeted mouse *Adamts1* gene in B16F1 cells, complemented with the proper control with a vector encoding a scramble sequence. Expression levels of *Adamts1* were severely downregulated upon interference (Supplementary Figure S2A–S2B). The characterization of these cells showed no relevant changes in the proliferation rate (Supplementary Figure S2C) although the migration capacity of interfered cells was increased upon seeding in different matrices (Supplementary Figure S2D). Being aware of this phenotypic characterization, we tested their tumorigenic properties with a similar syngeneic xenograft approach in WT and ATS1-KO mice as indicated in previous section. Now we included 3 different groups of cells: (i) B16F1 control cells, (ii) B16-shAts1 (with inhibited ADAMTS1), and (iii) B16-shSCR (scramble control). After 18 days all tumors were dissected and processed as previously. The analysis of final tumor weight revealed several findings (Figure 4A–4B). First we confirmed significant differences between tumors in WT and ATS1-KO mice for both control cells (B16F1 and B16-shSCR). Importantly, for tumors generated with inhibited B16-shAts1 cells, although following same tendency, they did not display significant differences between the WT and the ATS1-KO group. These results suggest a partial but minor contribution of ADAMTS1 derived from tumor cells. The evaluation of *Adamts1* gene expression within the tumors showed a similar percentage of inhibition than that described in the injected cells (approximately 50%) (Figure 4C).

In line with our previous studies, we performed a closer characterization of tumor vasculature by Endomucin staining and a subsequent morphometric evaluation with Metamorph 7 software. In general these analyses confirmed a significant increase in vessel density in tumors of ATS1-KO mice, independently of the type of implanted cells, with *Adamts1* suppression or not (Figure 4D–4E). Also in a similar pattern, an opposite reduction of average vessel perimeter and vessel area was observed (Figure 4E and Supplementary Figure S2E). Total area covered by vessels remained invariable between WT and ATS1-KO mice despite the type of cell injected (Supplementary Figure S2E). Additional gene expression data of endothelial markers from these tumors also confirmed that alterations depend on the nature of the animal (WT versus ATS1-KO mice) and that the downregulation of tumor *Adamts1* did not significantly contribute (Supplementary Figure S2F).

Furthermore, in order to assess vascular functionality, we evaluated gene expression of hypoxia-related genes HIF1a and HIF2a (Supplementary Figure S3A), and tumor hypoxia by the Hypoxyprobe assay, as described above. Again, significant differences were only noted between the animals of different genotypes (WT versus ATS1-KO) but not among those who had altered levels of *Adamts1* in the tumor cells (Supplementary Figure S3B).

**Figure 5:**
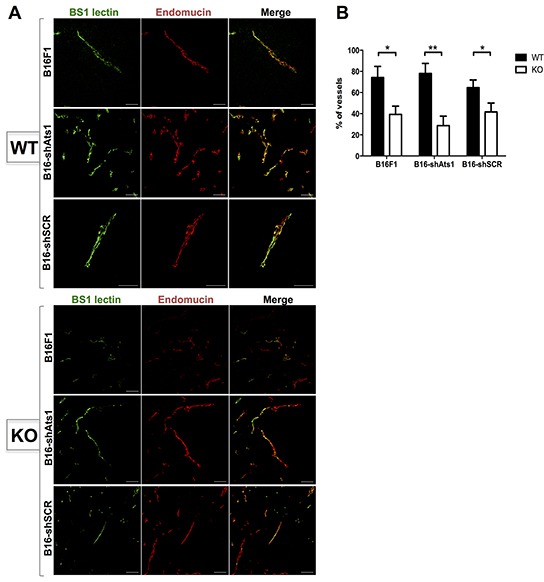
Evaluation of functionality of vasculature of tumors derived from control and modified B16F1 cells in WT and ATS1-KO mice **A.** Fluorescence microscopy images of tumor sections from different mouse groups, showing positive signal of Bs1 lectin (green), Endomucin immunostaining (red), and merge. **B.** Graph representing the quantification (in percentage) of the number of vessels that showed a colocalization of Bs1 lectin and Endomucin signals within each tumor sections. (Number of animals in this experiment: WT/B16F1, n=4; ATS1-KO/B16F1, n=4; WT/B16-shAts1, n=7; ATS1-KO/B16 shAts1, n=10; WT/B16-shSCR, n=6; ATS1-KO/B16-shSCR, n=8). Bars show mean values ± SEM. Statistical analyses show unpaired Student's t test. (*, p < 0.05; **, p < 0.01).

In this set of experiments we included a different approach to estimate functionality, as it is the intravenous injection of Bs1 lectin-FITC previous to sacrifice [27]. Subsequent microscopic evaluation of Endomucin-stained tumor sections revealed that a fewer percentage of vessels displayed colocalization of both molecules in tumors from ATS1-KO mice. These results confirmed the dysfunctionality of their vessels, in clear contrast to the WT group (Figure 5A–5B). Nevertheless, and consistently with our previous findings, quantification of fluorescence colocalization between groups of animals implanted with control B16F1 and interfered B16-shAts1 cells showed no significant differences (Figure 5A–5B). All these data together reinforce the notion that stroma-derived but not tumor ADAMTS1 supported tumor development through a vascular-dependent mechanism.

### Metastasis assays revealed a relevant contribution of host ADAMTS1 but not of tumor-derived ADAMTS1

**Figure 6:**
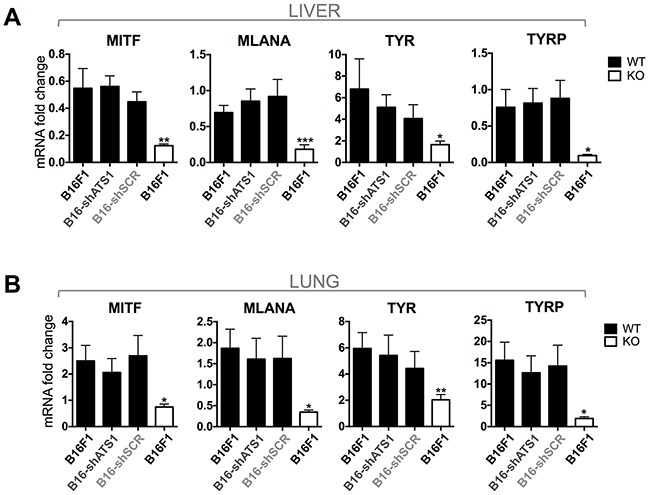
Gene expression analysis of melanoma markers (MITF, MLANA, TYR, TYRP) **A.** Graphs representing gene expression of the indicated genes in liver of WT and ATS1-KO mice. Animals were previously injected with control and modified B16F1 cells. In the case of WT mice (black bars), they were intravenously injected with non-modified cells (B16F1) (n=6), interfered shATS1 cells (B16-shATS1) (n=7), and scramble control cells (B16-shSCR) (n=5). Additionally, ATS1-KO mice (white bars) were injected with non-modified B16F1 cells (n=6). **B.** Graphs representing gene expression of the indicated genes in lung of WT and ATS1-KO mice. Groups are as specified for panel A. Bars show mean values ± SEM (*, p < 0.05; **, p < 0.01; ***, p < 0.001).

Some existing reports claim a pro-metastatic role of ADAMTS1 even with different experimental approaches [6, 7, 22, 28]. In our study we took advantage of the ATS1-KO mice to determine the contribution of stromal *Adamts1* in the metastatic cascade and, in addition, we included tumor cells with down-regulated *Adamts1*. We performed intravenous tail injection of control B16F1 and modified B16-shAts1 and B16-shSCR melanoma cells used in previous sections. The B16F1 cell model, although metastatic, produces a low number of macrometastasis. This property was positively considered in our research to avoid the potential masking of significant differences if the metastatic process is extremely active, as occurs with other cell models. A first microscopic evaluation of organs allowed us to visualize some metastatic nodules in the liver and lung of WT mice but almost none in the ATS1-KO animals (Supplementary Figure S4A–S4B). Furthermore, to obtain a more objective and quantitative measurement of micrometastasis, we assessed the gene expression of melanoma-related genes (MITF, MLANA, TYR and TYRP) [29] in RNA extracts from lungs and livers of experimental groups (Supplementary Figure 4C). qPCR analyses revealed strong differences between WT and ATS1-KO animals in both organs (Figure 6A–6B), in accordance with the visualization of metastatic nodules. Importantly, not significant differences were observed when inhibited B16-shAts1 cells were injected in the WT group (Figure 6A–6B). Again, a clear impairment of tumor progression, in this case affecting the metastatic process, appeared dependent of the presence of *Adamts1* in the stroma. However not major relevance was found for tumor-derived *Adamts1*.

### Stromal ADAMTS1 is induced in tumors and contributes to vascularization and macrophage infiltration during tumor progression

**Figure 7:**
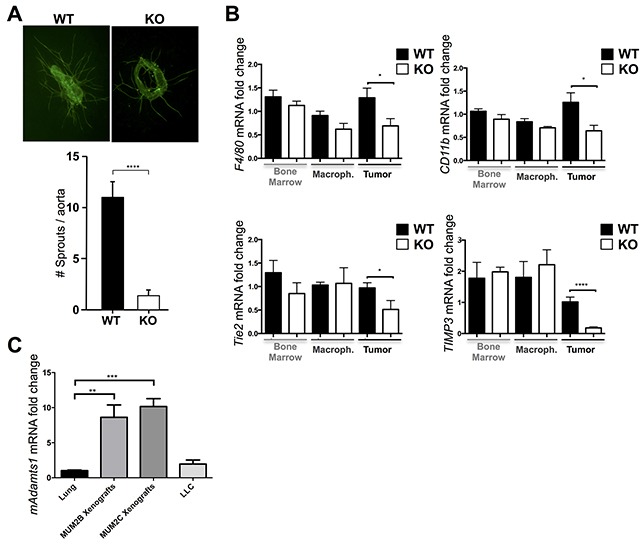
Contribution of ADAMTS1 to vascularization and macrophage infiltration and induction of its expression in stromal compartments **A.** Representative images (5x magnification) of *ex vivo* aortic rings (day 6) from WT and ATS1-KO mice, stained for FITC-BS1 Lectin to visualize sprouts. Accompanying graph represents the quantification of sprouts from aortic rings of WT (n=52) and ATS1-KO (n=69) animals. **B.** Graphs representing gene expression analyses of various macrophage-related markers (F4/80, CD11b, Tie2, TIMP3) in bone marrow, macrophages derived from bone marrow, and B16F1 tumors from WT and ATS1-KO mice. **C.** Graph representing gene expression of mouse *Adamts1* in lungs (n=5), MUM2B xenografts (n=8), MUM2C xenografts (n=21), and LLC cells (n=5). All statistical analyses show unpaired Student's t test (*, p < 0.05; ***, p<0.001; ****, p < 0.0001).

Given these results, in order to evaluate the contribution of stromal elements during tumor progression, we considered both vascular and inflammatory components in WT and ATS1-KO mice. First, to assess whether the dysfunctional vasculature observed in tumors in the ATS1-KO mice was associated to an anomalous angiogenic activity of their vessels, we performed *ex vivo* VEGF-induced aortic ring assays from WT and ATS1-KO animals. These analyses revealed that aortic ring sprouting was significantly reduced in aortas from ATS1-KO mice in comparison with WT controls (Figure 7A). These observations confirmed the relevance of ADAMTS1 expression during sprouting, in agreement with earlier results in 3D-collagen assays [15]. In addition, our immunocompetent B16F1 tumor model allowed us to investigate macrophage infiltrates, as another recognized and relevant constituent of the host stroma during tumor progression. With this goal we analyzed the gene expression of relevant macrophage-related molecules (F4/80, CD11b, Tie2 and TIMP3) [30, 31] in tumors (Figure 7B). In parallel, to know the basal status of our experimental mice, we tested the expression of these molecules in bone marrow extracts and macrophages derived from bone marrow of non-challenged mice. Whereas our qPCR assays did not display differences between the bone marrow and bone marrow-derived macrophages of WT and ATS1-KO, the analysis of tumor extracts did show a significant decrease in macrophage-related markers in the ATS1-KO group (Figure 7B) revealing an altered infiltration of macrophages probably due to the dysfunctionality of the vasculature.

To explore how stromal *Adamts1* is regulated following tumor induction, we approached the generation of human melanoma xenografts in immunodeficient mice. Importantly, these studies provided us the opportunity to determine the specific gene expression of human and mouse compartments (tumor and stroma, respectively) using species-specific primers. With this purpose we obtained xenografts with two uveal melanoma cell lines, MUM2B and MUM2C, which different endogenous levels of ADAMTS1 were previously revealed [5]. As the levels of mouse *Adamts1* transcripts have been found at low levels in a wide variety of organs [32] (and our unpublished results), we used lung tissue and the murine LLC cell line to normalize these results. Very significantly, mouse *Adamts1* expression was upregulated in both types of xenografts, independently of the endogenous levels of the protease in the human melanoma cells (Figure 7C). These results also supports a main role of ADAMTS1 provided by the host microenvironment as a relevant player during tumorigenesis.

## DISCUSSION

The current report reveals that the loss of ADAMTS1 in the host stroma leads to the impairment of tumor progression and development of metastasis. The use of a syngeneic tumor model with the ADAMTS1 KO mice, in combination with human xenografts and ex-vivo studies, allowed us to discriminate between the functional contribution of ADAMTS1 provided by tumor or stromal elements. Our findings also showed that the actions of ADAMTS1 affect functional neovascularization in the developing tumors with consequences for macrophage infiltration. It is likely that various mechanisms underlie these effects of ADAMTS1 according to its enzymatic activity and interaction with extracellular matrix components.

A relevant number of studies already implicated ADAMTS1 in tumor growth and metastasis although with different approaches (recently reviewed in [2]). For example, ADAMTS1 was found to be significantly over-expressed in clonal populations of highly metastatic cells to bone [33], and other authors have shown that its contribution to tumor development involved an induction of stroma remodeling [34]. More recently, the use of a tumor-prone model for mouse mammary carcinoma reported the requirement of *Adamts1* gene for invasion and progression [7]. Importantly this last report has been the only one, as far as we know, that included an *Adamts1*-deficient mouse [35] in a cancer-related study. The comparison with our model is compulsory and we must remark several distinctions. Firstly, Russell and colleagues [7] used the well recognized MMTV-PyMT transgenic mice [36] to evaluate mammary tumor progression. Since the absence of *Adamts1* occurs in all cells, both neoplasic and stromal, it is not possible to discriminate between the relevance of the protease secreted by tumor cells or by different stromal components. As for our approach, although we need to agree about the caveat that tumors do not develop in a spontaneous manner, the implantation of tumor cells in a genetically modified mouse remains as a powerful tool to unveil the specific contribution of singular compartments, namely tumor versus stroma. Remarkably our results also support a pro-tumorigenic and pro-metastatic activity of ADAMTS1, and we provided for the first time the finding that ADAMTS1 originated in the stroma contributes more significantly than ADAMTS1 derived from tumor cells. The use of a syngeneic model with genetically-modified animals has been described for a variety of extracellular molecules [37–42]. In this study we added the manipulation of our gene of interest in the implanted tumor cells, revealing its minor contribution, at least in this tumor model.

The evaluation of *Adamts1* gene expression in our different experimental groups also provided a critical observation. We considered very relevant the lack of differences in *Adamts1* expression between tumors generated in WT and in ATS1-KO animals (Figure 1C and 4C), although tumor progression was clearly affected (Figure 1B and 4B). In fact we even detected that *Adamts1* tumor expression suffered an induction in the ATS1-KO mice, probably related with its regulation by hypoxia. Such findings suggested that currently ongoing expression analyses of whole tumor samples might not provide the proper information to discard or to predict the malignancy of a neoplasia. With a similar perspective, an avalanche of reports on tumor heterogeneity are emphasizing the necessity to implement current clinical strategies [43]. Here we also highlight the importance to discriminate between stroma and tumor components.

Very interestingly, our recent results demonstrate that the basal expression of ADAMTS1 in stromal cells, such as endothelium and macrophage-related population, is induced in a tumor context. Such findings suggest a relevant role for this protease under stressing conditions. The identification of specific substrates in every situation is still a current and required focus on the extracellular matrix field.

The implication of ADAMTS1 (and other ADAMTS members) for various vascular-related phenomena has been a constant finding since the first functional studies, though the results are apparently contradictory, with both pro- and anti-angiogenic features (reviewed in [44]). Still there is necessity to investigate the extracellular components of every cellular model in order to unveil this complex picture. For example, multiple abnormalities have been described in the basement membrane surrounding tumor vessels compared with normal vasculature [45], such as distinct thickness, reduced pericyte coverage, but also altered processing of basic components [4]. In this work we found that the lack of ADAMTS1 precludes endothelial sprouting in the aortic ring assay. Furthermore, tumor vasculature in ATS1-KO mice appeared dysfunctional, showing strong hypoxic areas.

Finally, our finding that infiltration of macrophages is challenged in tumors in the absence of stromal ADAMTS1 provokes a novel perspective for future research. In one side the reciprocal interaction, between the vasculature and the specialization of macrophages, deserves a major effort. Furthermore, the link of this observation with the current and successful advances in immunotherapies to fight cancer definitively emphasizes the importance to get deeper in these studies.

## MATERIALS AND METHODS

### Vectors, lentiviral production, and transduction

Knockdown vectors containing shRNAs to inhibit ADAMTS1 expression were purchased from Sigma (MISSION, St. Louis, MO, USA). Lentiviral particles pseudotyped with the VSV-G protein were generated on HEK293T cells using a standard calcium-phosphate transfection protocol. Mouse B16F1 melanoma cells were infected overnight. The following day, the viral supernatant was removed and transduced B16F1 cells were washed with media and allowed to expand. Media containing puromycin 1μg/ml was added to cultures and selection was carried out for 2 weeks. ADAMTS1 downregulation was tested by qPCR and western blot analyses (Supplementary Figure S2A–S2B).

### Cell culture

Mouse tumor cells B16F0, B16F1, Lewis Lung Carcinoma (LLC), and brain immortalized endothelial cells (BEND), were cultured in DMEM supplemented with 10 % fetal bovine serum (FBS), and 1 % penicillin-streptomycin. Mouse aortic primary endothelial cells (MAEC) from C57BL/6 mice (Cell Biologics Inc, Chicago, IL) were cultured in complete mouse endothelial cell culture medium (M1168 Kit, Cell Biologics Inc). Human umbilical vein endothelial cells (HUVECs) (Lonza, Switzerland) were cultured in EGM-2 medium. Human embryonic kidney (HEK293T) cells were grown in DMEM supplemented with 10 % FBS, 1 % penicillin-streptomycin and 2 mM L-glutamine. All cells were maintained at 37°C under 5 % CO_2_ atmosphere and 95 % humidity.

### Tumor xenograft and metastasis assays

All mice were kept in the Centro de Investigaciones Biomédicas-UGR Animal Facility under pathogen-free conditions and according to institutional guidelines. For the generation of syngeneic tumors, 1×10^6^ cells in 200 μl of PBS were subcutaneously injected in the right flank of C57Bl/6 mice from wild type and *Adamts1* KO (ATS1-KO) genotypes [46]. Tumor growth was monitored up to 18 days. All animals were sacrificed following proper ethical and institutional guidelines. Tumors were dissected and processed for further analysis.

For the metastasis assay, 3×10^5^ cells in 100 μl of PBS were intravenously injected through the tail vein of C57Bl/6 mice, also from wild type and ATS1-KO genotypes. These mice were under observation for 13 days and then they were sacrificed and various organs were properly dissected for further analysis.

Human melanoma xenografts were generated in Swiss Nude mice with human uveal melanoma cells MUM2B and MUM2C [5]. 1×10^6^ cells in 200 μl of PBS were subcutaneously injected in the right flank, and tumor growth was monitored during 18-25 days. Tumors were dissected and processed for gene expression analysis.

### Immunohistological analysis and vasculature characterization

A morphometric analysis of vessels was obtained by immunostaining with a monoclonal rat anti-mouse Endomucin antibody (SC-65495, SCBT) in tumor sections, and images were captured with the AxioImager A1 microscope (Zeiss). Depending of the size of the section, up to 10 fields per tumor were captured. All the analyses were done as previously reported [27], in this case using Metamorph 7 software (Molecular Devices, California, USA). Grey scale images of endomucin-stained sections are required. Previous to quantification, the operator establishes an optimal threshold (to discard non-specific signals) that it is maintained through the complete study. Accordingly, the application identifies positive signals and quantifies the number of positive units (number of vessels), and the area and perimeter of positive units. Simple mathematical functions estimate the average and total area, average and total perimeter, and additional parameters.

To measure hypoxia we followed the instructions of the Hypoxyprobe-1 kit (Hypoxyprobe Inc., Burlington, Mass, USA). Accordingly, 60 mg/kg pimonidazole hydrochloride were injected intraperitoneally into tumor-bearing mice that were sacrificed 30 minutes later. Tumor tissues were properly dissected and embedded in paraffin. Immunofluorescence staining using an antipimonidazole monoclonal FITC antibody was performed according to the manufacturer's instructions. Images were captured with a LSM710 (Zeiss) and quantification was done using ZEN 2010B SP1 software (Zeiss).

To assess vascular viability we injected intravenously a group of tumor-bearing mice with 100 μl of FITC-conjugated *Bandeira simplicifolia (Bs1)* lectin (50 μg/ml). 5 minutes later animals were anesthetized with intraperitoneal administration of ketamine (100 mg/kg) and xylazine (10 mg/kg). In sedated mice we administered 500 μl of PFA 4 % intracardially in the left ventricle to fix the lectin.

### Aortic ring assay

Aortas from different animal backgrounds were extracted, cleaned from fat and connective tissue and embedded in a matrix of collagen I for 1 h at 37°C to solidify, as previously described [47]. Optimem media supplemented with 2,5 % FBS and 30 ng/ml VEGF was added to rings and they were maintained at 37°C and 8 % CO_2_. Media was refreshed every three days. After sprouting, the rings were fixed for staining with 4 % PFA for 1 h. Tips from sprouts were counted at day 10.

### Bone marrow extraction and macrophage differentiation

Wild type and ATS1-KO C57Bl/6 mice aged between 8 and 12 weeks were sacrificed and dissected following the proper ethic guidelines. Their femur and tibia were extracted from both legs followed by the cut of the boneheads, leaving the bone cane open. A 23G syringe with PBS was used to flush the bone marrow out of the bone to continue with the disaggregation into single cells. The resulting extract was filtered using a 100 μm cell strainer followed by a 300 g spin during 5 minutes at RT. The resulting cell suspension was counted and seeded at a concentration of 4×10^5^ cells/ml in a 10 cm non-treated tissue culture plate, containing DMEM 10 % FCS, 1 % P/S and 10 ng/ml mCSF (Peprotech 300-25). Culture was maintained for 7 days with a media change at day 3. Flow cytometry with CD11b Antibody (553311, BD Pharmigen) was performed at day 7 to check the enrichment in macrophage populations. At different stages, bone marrow and macrophage lysates were obtained for RNA or protein extraction.

### Quantitative RT-PCR and statistical analysis

Total RNA was extracted from tumor biopsies and tissues using the NucleoSpin RNAII kit (Macherery-Nagel). cDNA was synthesized with iScript cDNA Synthesis Kit (Bio-Rad). qPCR reaction was performed in a 7900HT PCR machine (Applied Biosystems) using Fast SYBR green master mix (Applied Biosystems). qPCR representations show the 2^(−ΔΔCt) value using actin, β2 microglobulin and 18S RNA as reference genes. A geometric mean of the selected housekeeping genes was used for normalization. Values show mean ± standard error of the mean (SEM). Statistical analyses were performed by unpaired Student's t test using GraphPad Prism software. Differences were considered statistically significant at p < 0.05.

### Western blot analysis

Conditioned medium from cells was clarified and concentrated with StrataClean resin (Stratagene) as previously described [12]. Total protein from tumor samples and cell lysates was extracted using RIPA buffer containing 1 mM PMSF, 1 μg/ml aprotinin and 10 μg/ml leupeptin. Proteins were resolved by SDS-PAGE and transferred to PVDF membranes (BioRad). Membranes were blocked with 5 % low-fat milk and incubated with the monoclonal mouse anti-human ADAMTS1 antibody (AF5867, R&D Systems). After incubation with the appropriate secondary peroxidase-conjugated antibody, signal was detected with the SuperSignal West Dura Chemiluminescence Kit (Pierce) in an ImageQuant LAS4000 (GE Healthcare Life Sciences).

## SUPPLEMENTARY FIGURES


