# Supplementary material for: Stroma-derived but not tumor ADAMTS1 is a main driver of tumor growth and metastasis
Source: Oncotarget. 2016 Apr 22;7(23):34507–19. doi: 10.18632/oncotarget.8922 (PMC5085172; doi:10.18632/oncotarget.8922)
Supplement: Supplementary file 1 [file oncotarget-07-34507-s001.pdf]

Stroma-derived but not tumor ADAMTS1 is a main driver of tumor growth and metastasis

SUPPLEMENTARY FIGURES

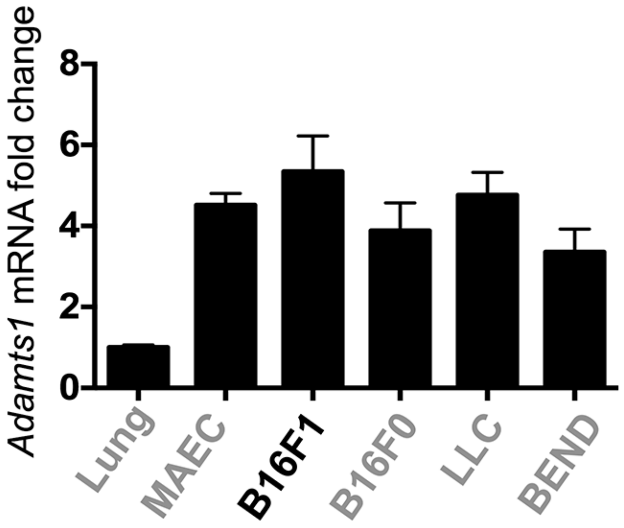

Supplementary Figure S1: Graph representing Adamts1 gene expression in different tumor and primary mouse cell lines in culture, compared with mouse lung. Bars show mean values ± SEM.

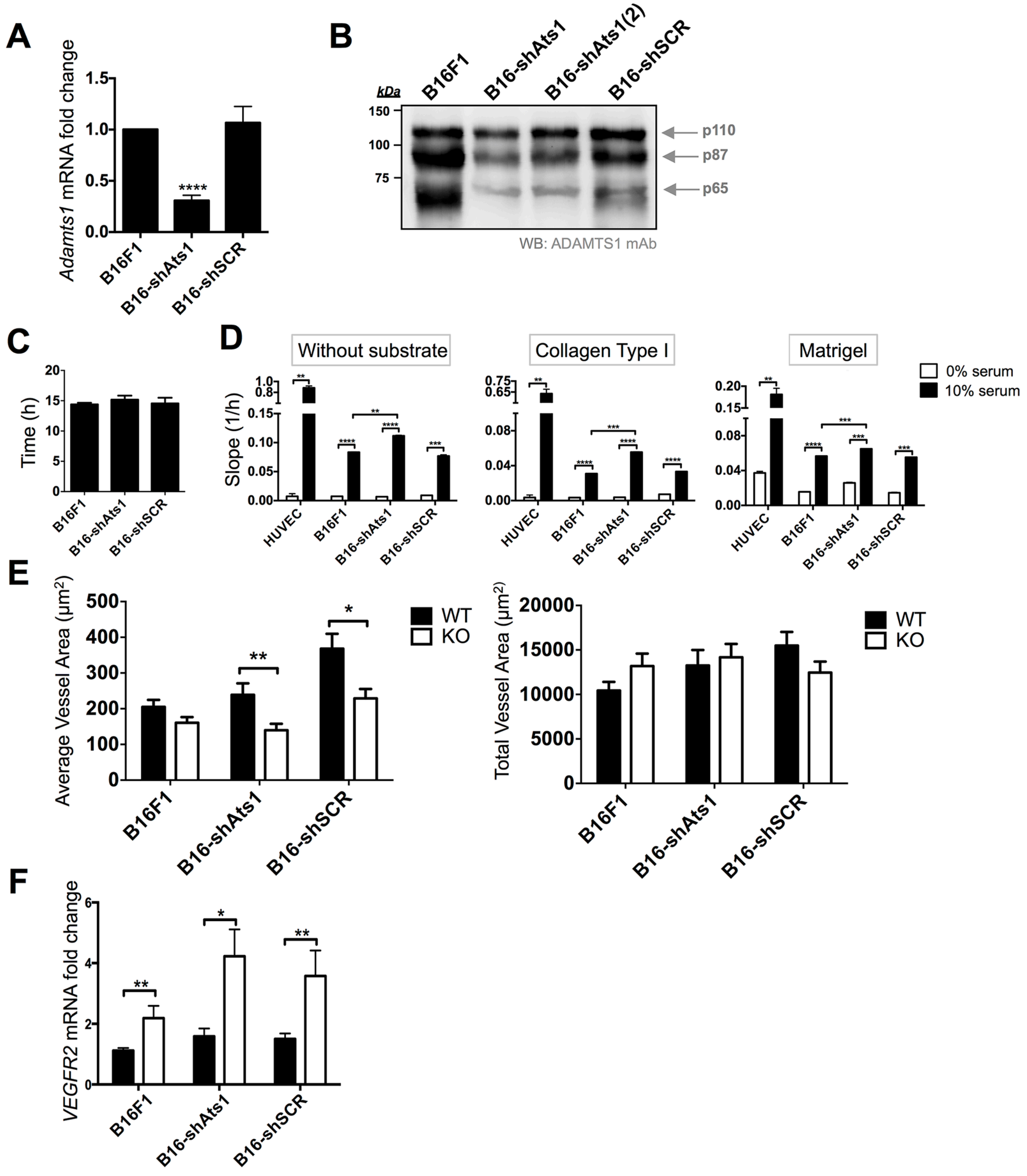

Supplementary Figure S2: Studies with control and modified B16F1 cells, and additional characterization of vasculature of tumors derived from control and modified B16F1 cells in WT and ATS1-KO mice. A. Graph representing Adamts1 gene expression in control B16F1 cells, shADAMTS1 targeted cells (B16-shAts1) and the non-target shRNA control (B16-shSCR). Bars show mean values ± SEM. **B.** Protein analysis by Western-Blot of ADAMTS1 in control and modified B16F1 cells, as mentioned in panel B. Two different shADAMTS1-inhibited cell populations are showed. **C.** Graph representing doubling time of control and modified B16F1 cells, showing that the downregulation of ADAMTS1 does not provoke any effect. **D.** Graphs representing the migration rate in different substrates, determined in an XCelligence platform, of control and modified B16F1 cells, also including HUVEC as a positive control for the assay. **E.** Graphs representing results from the morphometric analyses of vasculature (Metamorph 7 software) of tumors from different groups as indicated. These analyses include average vessel area (µm^2), and total vessel area (µm^2). **F.** Graphs representing gene expression evaluation of vascular related VEGFR2 in tumors from different groups as indicated. Bars show mean values ± SEM. (*, $p < 0.05$; **, $p < 0.01$; ***, $p < 0.001$; ****, $P<0.0001$).

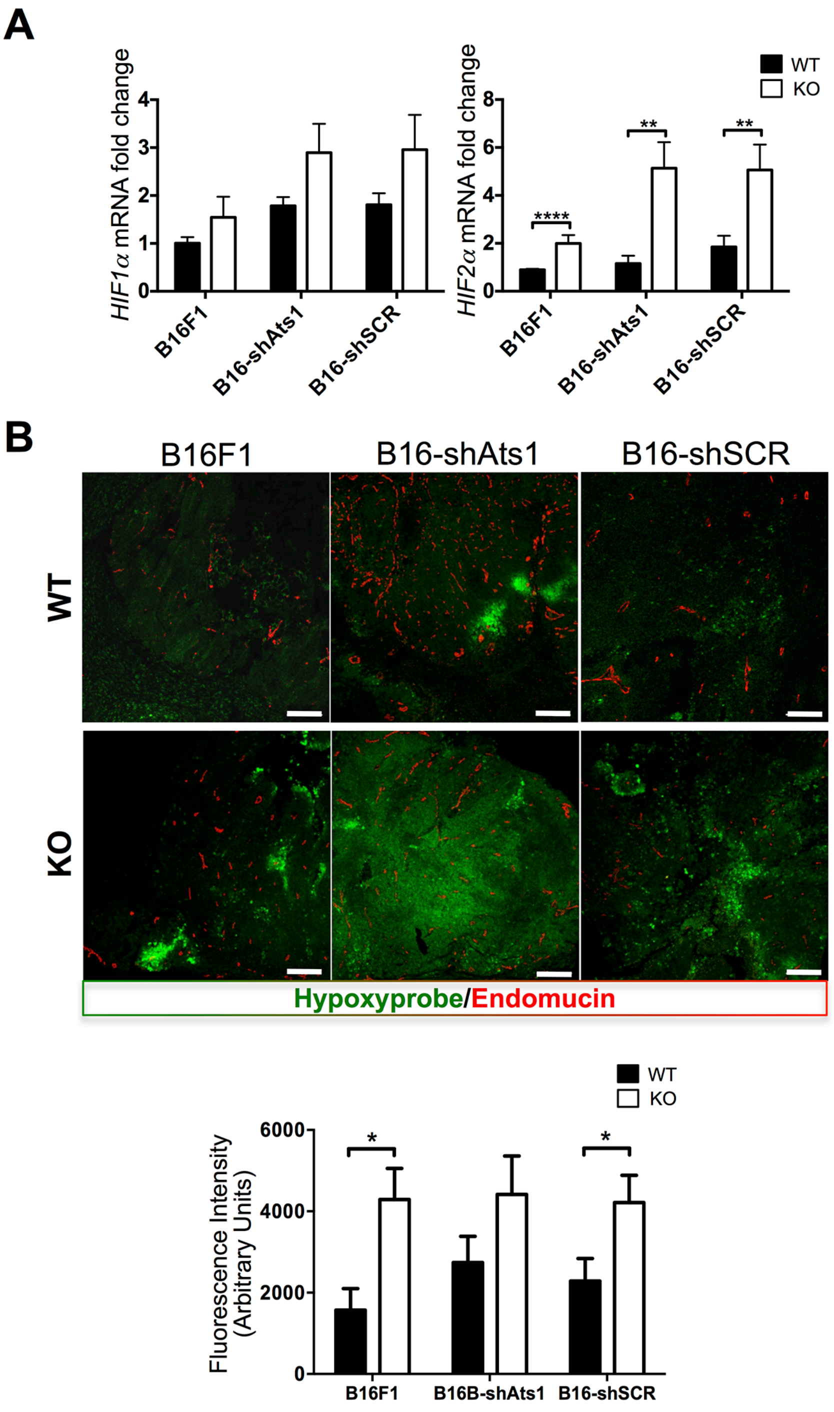

Supplementary Figure S3: Additional characterization of hypoxia of tumors derived from control and modified B16F1 cells in WT and ATS1-KO mice. A. Graphs representing gene expression evaluation of hypoxia-related genes (HIF1α and HIF2α) in tumors from different groups as indicated. Bars show mean values ± SEM. (**, $p < 0.01$; ****, $P<0.0001$). **B.** Representative images from confocal microscopy showing immunolabelling of Hypoxyprobe (green) and Endomucin (red) in tumors generated with control and modified B16F1 cells in WT and ATS1-KO mice. Graph in the right side represents a quantification of fluorescence intensity in tumor sections from different groups as indicated. (*, $p < 0.05$; **, $p < 0.01$; ****, $p < 0{,}0001$).

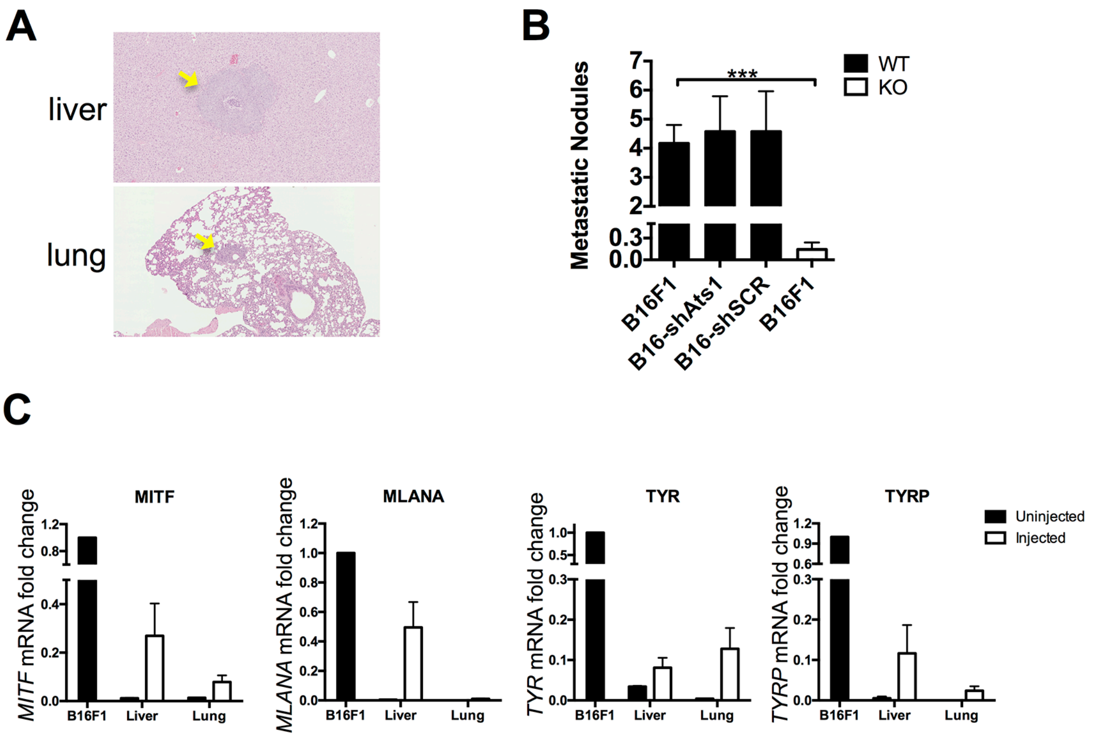

Supplementary Figure S4: Evaluation of B16F1-derived metastasis in liver and lung. Control and B16F1-injected mice were sacrificed and their organs were properly dissected and processed for RNA extraction and paraffin sections. **A.** Representative brightlight microscopic images of metastatic nodules found in the liver and lung of B16F1-injected mice. To visualize these nodules, paraffin sections were stained with Hematoxylin-eosin. **B.** Graph representing the quantification of visible metastatic nodules in whole liver sections of WT and ATS1-KO mice injected with the indicated cells (control and modified B16F1). Bars show mean values ± SEM. **C.** Graphs representing gene expression analysis of melanoma markers (MITF, MLANA, TYR, TYRP) in liver and lung of control uninjected and B16F1-injected WT mice. B16F1 cells were used as a positive control.
